# TNF inhibitors in the treatment of uveitis in the course of juvenile idiopathic arthritis

**DOI:** 10.1186/1546-0096-12-S1-P237

**Published:** 2014-09-17

**Authors:** Violetta Opoka-Winiarska, Jacek Postępski, Agnieszka Korobowicz-Markiewicz, Anna Bodajko-Grochowska, Aleksandra Szabat, Andrzej Emeryk

**Affiliations:** 1 Department of Pediatric Pulmonology and Rheumatology, Medical University of Lublin, Poland, Lublin, Poland

## Introduction

Uveitis is the most common extraarticular complication of juvenile idiopathic arthritis (JIA). TNF inhibitors are a promising new therapeutic option in the treatment of chronic uveitis in JIA.

## Objectives

The aim of this study was to evaluate the efficacy of TNF inhibitors in children with chronic uveitis in the course of JIA.

## Methods

Data of 8 children with JIA and chronic uveitis treated with TNF inhibitors between 2010 and 2013 were analysed retrospectively. The clinical subtypes of JIA were: polyarticular – 2 patients (pts), oligoarticular - 6 pts (according to the ILAR criteria). 5 children were ANA positive. Clinical effectiveness assessment included JIA outcome parameters (PhGA, PaGA, CHAQ, ESR/CRP, number of joints with active arthritis; number of joints with limited range of motion) and ophthalmological examination.

## Results

In all (8) pts the first used biological drug was ETA after unsuccessful treatment with two synthetic DMARDs (including methotrexate) and systemic glucocortycoid (6 pts) and topical glucocortycoid (8 pts). The treatment of ETA was started after a mean 4 ± 3,7 years of disease, and mean 3,87±3,9 after uveitis was diagnosed. In all patients, remission of uveitis was achieved during ETA treatment. ETA treatment was terminated after at least 18-month remission on the drug (according to the Polish therapeutic program). 3 pts (43%) developed a uveitis exacerbation after termination of ETA and the treatment was restarted. In 2 pts due to exacerbation of uveitis, ETA was switched to adalimumab with improvement. In all children systemic and topical glucocorticoids were terminated.

## Conclusion

Anti-TNF therapy is effective in JIA patients with chronic uveitis. In case of active disease it is necessary to switch to another biological agent. Many patients developed a disease exacerbation after anti-TNF termination, reintroduction of therapy was needed.

## Disclosure of interest

None declared.

